# Antioxidant and Cytotoxic Effects of Crude Extract, Fractions and 4-Nerolidylcathecol from Aerial Parts of *Pothomorphe umbellata* L. (*Piperaceae*)

**DOI:** 10.1155/2013/206581

**Published:** 2012-12-19

**Authors:** Andrey P. Lopes, Bianca S. Bagatela, Paulo C. P. Rosa, Dhammika N. P. Nanayakkara, José Carlos Tavares Carvalho, Edson L. Maistro, Jairo K. Bastos, Fábio F. Perazzo

**Affiliations:** ^1^Departamento de Ciências Exatas e da Terra, Instituto de Ciências Ambientais, Químicas e Farmacêuticas, Universidade Federal de São Paulo, São Nicolau Street 210, 09972-270 Diadema, SP, Brazil; ^2^National Center for Natural Products Research, Research Institute of Pharmaceutical Sciences, The University of Mississippi, Oxford, MS 38677, USA; ^3^Laboratório de Pesquisa em Fármacos, Universidade Federal do Amapá, 68902-280 Macapá, AP, Brazil; ^4^Departamento de Fonoaudiologia, Faculdade de Filosofia e Ciências, Universidade Estadual Paulista, 17525-900 Marília, SP, Brazil; ^5^Departamento de Ciências Farmacêuticas, Faculdade de Ciências Farmacêuticas de Ribeirão Preto, Universidade de São Paulo, 14040-903 Ribeirão Preto, SP, Brazil

## Abstract

The crude ethanolic extract from aerial parts of *Pothomorphe umbellata* L. (*Piperaceae*) and fractions obtained by partitions sequentially among water-methanol, methylene chloride, and ethyl acetate, as well as the major constituent, 4-nerolidylcatechol, were, respectively, evaluated and evidenced for antioxidant and cytotoxic effects through fluorometric microplate and microculture tetrazolium assays in HL-60 cells. The crude ethanolic extract demonstrated the preeminent antioxidant activity (IC_50_ = 1.2 **μ**g/mL) against exogenous cytoplasmic reactive oxygen species, followed by the water-methanolic (IC_50_ = 4.5 **μ**g/mL), methylene chloride (IC_50_ = 5.9 **μ**g/mL), ethyl acetate (IC_50_ = 8.0 **μ**g/mL), 4-nerolidylcatechol (IC_50_ = 8.6 **μ**g/mL), and the sterol fractions (IC_50_ > 12.5 **μ**g/mL). Vitamin C, the positive control used in this assay, presented IC_50_ value equivalent to 1.7 **μ**g/mL. 4-Nerolidylcatechol (IC_50_ = 0.4 **μ**g/mL) and methylene chloride fraction (IC_50_ = 2.3 **μ**g/mL) presented considerable cytotoxicity probably because of the presence of an *o*-quinone, an auto-oxidation by product of the catechol. Polar compounds, present in the ethanol extract, appear to increase the solubility and stability of the major active constituent, acting synergistically with 4-nerolidylcatechol, improving its pharmacokinetic parameters and increasing significantly its antioxidant activity which, in turn, suggests that the aqueous-ethanolic extract, used in folklore medicine, is safe and effective.

## 1. Introduction

Plants belonging to *Piperaceae* family are reputed in the Indian Ayurvedic system of medicine and in folklore medicine of Latin America and West Indies for their medicinal properties [1]. *Piper umbellata*, former *Pothomorphe umbellata* L. (*Piperaceae*), known in Brazil as “caapeba” or “pariparoba,” is a shrubby plant which thrives spontaneously in moist and shady places from Amazon to the south of Brazil [2]. Its species, described in the first Brazilian Pharmacopoeia [3], has been assigned to different pharmacological activities currently verified, such as an anti-inflammatory and analgesic [4], and antiulcer and gastroprotective [5], antimalarial [6] and antioxidant [7].

Free radicals and reactive oxygen species have been proven to mediate processes involved in the pathogenesis of a variety of ailments including inflammation, cancer, diabetes, liver cirrhosis, cardiovascular diseases, and premature aging [8, 9]. Free radicals, with their unpaired electrons, can attack and damage almost any molecule found in the body. They are so active that, once formed, bind to different compounds in seconds. Therefore, they can deliver their nonpaired electron or capture another molecule electron to form a pair. Then, radicals become stable and, consequently, the molecule previously attacked becomes a radical, which promotes a chain reaction that act destructively in human tissues [10]. 

Reactive oxygen species, in turn, are significantly present in biological processes of energy production and phagocytosis [11]. The main superoxide anions are (O_2_^−^), hydroxyl radical (OH^−^), nitric oxide (NO), hydrogen peroxide (H_2_O_2_), and lipid radical (L^−^). Among these, hydroxyl radical is more reactive in the induction of lesions in cellular molecules whilst hydrogen peroxide is sufficiently able to cross the nuclear membrane and cause damage to the DNA molecule [12]. Thus, effective and safe antioxidants acquired sustainably from the biodiversity can diminish the threat of free radicals and reactive oxygen species damage over lifetime [13]. 

Nowadays, attention is outlined to antioxidants originating from natural products which offer new treatment possibilities for diseases mediated by oxidative stress. Many enzymes and secondary metabolites of higher plants have been demonstrated in *in vitro *and *in vivo* experiments to be capable of protecting tissues against oxidative stress through the free radicals and oxygen reactive species inhibition or capture [14], besides its consumption has been associated with the decreased risk of degenerative diseases occurrence [15]. Brazil has a flora that is extremely rich in medicinal plants with immense potential for supplying these antioxidant agents. Among these plants, members of *Piperaceae* family are rich in phenolic compounds [16], in particular, *Pothomorphe umbellata* L.

In relation to the antioxidant activity of *Pothomorphe umbellata* L., its crude extract has been evaluated by *in vitro* assays, using as model the self-oxidation of mouse brains, and such activity was partly attributed to the presence of 4-nerolidylcathecol [17], a phenolic compound, isolated from the vegetable roots and leaves [18]. Commonly, the defensive effects of natural antioxidants are related to the presence of phenolic compounds [19]. However, in another *in vitro* assay, the *Pothomorphe umbellata *L. crude extract showed an antioxidant potential significantly larger than the one of this isolated compound, suggesting the presence of additional compounds with antioxidant activity [20]. 

Based on these data, this study was undertaken to evaluate and evidence the antioxidant activity of crude ethanolic extract, sterol fraction, and 4-nerolidylcathecol from aerial parts of *Pothomorphe umbellata *L. as well as its cytotoxic effect to investigate and ascertain toxic or antiproliferative actions of these test samples in HL-60 cells and, then, to verify if the crude aqueous-ethanol extract prepared from the aerial parts of *Pothomorphe umbellata *L. and widely used in folklore medicine is, in fact, secure and efficient.

## 2. Materials and Methods

### 2.1. Plant Material

Leaves from *Pothomorphe umbellata* L. were collected in March 2011 on the campus of the University of São Paulo in Ribeirão Preto, São Paulo, Brazil. The botanical identification of the leaves was made by Prof. Pedro Melillo de Magalhães and a voucher sample with registration number UEC127123 was deposited in the herbariumof the Botany Departmentof State University of Campinas, Campinas, Brazil.

### 2.2. Preparation of Extract and Fractions

The crude ethanolic extract and fractions from *Pothomorphe umbellata* L. were prepared as described preliminarily [4]. The ethanolic extract was suspended in methanol-water (9 : 1) and extracted with methylene chloride (CH_2_Cl_2_) and ethyl acetate (EtOAc), in sequence, to furnish methanol (MeOH), CH_2_Cl_2_, and EtOAc fractions. The CH_2_Cl_2_ was separated into hexane soluble and insoluble parts. The hexane insoluble part, analyzed by GC-MS, was found to be composed of *β*-sitosterol, campesterol and stigmasterol. 

### 2.3. Isolation and Identification of 4-Nerolidylcatechol

The 4-nerolidylcatechol was isolated as reported previously [18] and properly identified by nuclear magnetic resonance. NMR spectra were obtained on Bruker DPX 400 MHz apparatus, operating at 400 MHz for ^1^H NMR and at 100 MHz for ^13^C NMR. The samples were dissolved in deuterated chloroform (Aldrich).

### 2.4. Microplate Assay for Oxidative Products Detection Using DCFH-DA in HL-60 Cells

A fluorometric microplate assay [21] was established for the detection of oxidative products using 2′,7′-dichlorofluorescin-diacetate (DCFH-DA) in human promyelocytic leukemia cells (1 × 10^6^ HL-60 cells/mL, ATCC) which were suspended in RPMI 1640 medium with 10% FBS and antibiotics at 37°C in 5% CO_2_: 95% air. Then, 125 *μ*L of the cell suspension were added into each well on 96-well microtiter plates. After treatment with different concentrations of the test material for 30 minutes, the cells were stimulated with 100 ng/mL of phorbol 12-myristate 13-acetate (PMA, Sigma) for 30 minutes. Then, the cells were incubated for 15 minutes after the addition of 5 *μ*g/mL of DCFH-DA (Molecular probes). The ability of the test materials to inhibit exogenous cytoplasmatic reactive oxygen species-catalyzed oxidation of DCFH-DA in HL-60 cells was measured by PMA treated control incubations with and without the test materials. The levels of DCFH-DA were measured using a CytoFluor 2350 fluorescence measurement system (Millipore) with an excitation wavelength at 485 nm (bandwidth 20 nm) and an emission at 530 nm (bandwidth 25 nm). Vitamin C and trolox were used as the positive controls in this assay.

### 2.5. XTT Assay for Cytotoxicity in HL-60 Cells

Cellular growth in the presence or absence of experimental agents was determined using the previously described microculture tetrazolium assay [22]. The tetrazolium reagent (XTT) was designed to yield a suitably colored, aqueous-soluble, nontoxic formazan upon metabolic reduction by viable cells. After sample (25 *μ*L) exposure on cells for 48 hours, the XTT assay was performed. Briefly, rapidly growing cells were harvested, counted, and inoculated at the appropriate concentrations (100-*μ*L volume) into 96-well microtiter plates using a multichannel pipet. Accordingly, 25 *μ*L of XTT-PMS solution (1 mg/mL XTT solution supplemented with 25 *μ*M of PMS) was added to HL-60 cells (2 × 10^4^ cells in 225 *μ*L of medium) into each well on the microtiter plates. After incubation for 4 hours at 37°C, absorbance at 450 nm was measured by a microplate reader (reference absorbance at 630 nm). Vitamin C and trolox were used as the positive controls in this assay.

### 2.6. Statistical Analysis

The statistical analyses were established using Analysis of Variance (ANOVA) followed by the Tukey-Kramer multiple comparison test [23]. Results with *P* < 0.05 were considered to be significant. Data are expressed as mean ± S.D.

## 3. Results

### 3.1. Isolation and Identification of 4-Nerolidylcathecol

Analysis of the ^1^H NMR spectrum evidences the presence of aromatic group by chemical shift and coupling constants relative for the three hydrogens, H-3 at *δ* 6.87 ppm (d,  *J* = 2 Hz), H-5 at *δ* 6.75 ppm (dd,  *J* = 8.4 Hz and  *J* = 2 Hz), and H-6 at *δ* 6.80 ppm (d,  *J* = 8.4 Hz). The data from ^13^C NMR spectrum permitted the verification of aliphatic chain of catechol and nerolidyl groups by the presence of two methyl groups attached to carbon sp^2^ not containing hydrogen (*δ* 124.4 ppm and *δ* 124.6 ppm). The methyl hydrogens linked to sp^2^ carbon (Me11′) presented chemical shift between *δ* 1.5 ppm and *δ* 1.7 ppm (*singlet*), as well as protons of the methyl group linked to sp^3^ carbon with *δ* 1.3 ppm. The data obtained are similar to data presented by Kijjoa et al. [18] suggesting that 4-nerolidylcathecol has been appropriately isolated and identified.

### 3.2. Microplate Assay for Oxidative Products Detection Using DCFH-DA in HL-60 Cells

Test samples and the positive controls, vitamin C and trolox, were evaluated for the inhibition of exogenous cytoplasmic reactive oxygen species-catalyzed oxidation using 2′,7′-dichlorofluorescin-diacetate (DCFH-DA) in human promyelocytic leukemia cells (HL-60 cells). IC_50_ concentrations were established for the purpose of verifying *Pothomorphe umbellata* L. antioxidant effect which is displayed in Table 1. 

The crude ethanolic extract from aerial parts of *Pothomorphe umbellata *L. demonstrated the best antioxidant activity (IC_50_ = 1.2 *μ*g/mL). This activity was higher than that observed for vitamin C (IC_50_ = 1.7 *μ*g/mL). The MeOH and EtOAc fractions presented, respectively, the lowest (IC_50_ = 4.5 *μ*g/mL) and the highest antioxidant activity (IC_50_ = 8.0 *μ*g/mL) among the assayed fractions, while the CH_2_Cl_2_ fraction demonstrated an intermediate effect (IC_50_ = 5.9 *μ*g/mL). All these fractions exhibited lower activity than the crude extract. 4-Nerolidylcatechol, the major constituent present in this species, which is recognized to have a noteworthy antioxidant potential, displayed an activity even lower (IC_50_ = 8.6 *μ*g/mL) in this assay. The sterol fraction did not present a significant antioxidant effect (IC_50_ > 12.5 *μ*g/mL), inhibiting the oxidative products formation by 28% at concentration equivalent to 62.5 *μ*g/mL.

### 3.3. XTT Assay for Cytotoxicity in HL-60 Cells

Test samples and the positive controls, vitamin C and trolox, were evaluated for cytotoxicity through cellular growth in the presence or absence of these experimental agents using the XXT-microculture tetrazolium assay in human promyelocytic leukemia cells (HL-60 cells). IC_50_ concentrations were established for the purpose of verifying *Pothomorphe umbellata* L. cytotoxic effect which is displayed in Table 2. 

4-Nerolidylcathecol demonstrated the preeminent cytotoxicity (IC_50_ = 0.4 *μ*g/mL) in HL-60 cells, followed by CH_2_Cl_2_ fraction (IC_50_ = 2.3 *μ*g/mL), crude ethanolic extract (IC_50_ = 5.3 *μ*g/mL), and sterol fraction (IC_50_ = 8.9 *μ*g/mL). The patterns of vitamin C and trolox had not evidenced cytotoxicity at the highest tested dose. However, 4-nerolidylcatechol and CH_2_Cl_2_ fraction demonstrated a significant cytotoxic effect.

## 4. Discussion

Analysis of the crude ethanolic extract and fractions from aerial parts of *Pothomorphe umbellata* L. indicated that most of the 4-nerolidylcatechol molecules and sterols are concentrated in the CH_2_Cl_2_ fraction. The major reason for the observed lesser activity of 4-nerolidylcatechol and the CH_2_Cl_2_ fraction in comparison with the crude ethanolic extract must be correlated to solubility and stability. These pharmacokinetic properties are closely related to the pharmacological effectiveness once the antioxidant efficacy depends on the ability of compounds to penetrate the cell membrane [13]. Then, the probable reason for the lower activity of 4-nerolidylcathecol and sterol fraction, compared with the crude extract, should be correlated to solubility and stability. Therefore, other compounds, present in the crude extract, must act synergistically with 4-nerolidylcathecol, improving its pharmacokinetic parameters and increasing significantly its antioxidant activity.

Pure 4-nerolidylcatechol is labile in ambient light, air, and room temperatures [24] and, thus, tends to undergo rapidly an autooxidation to an *o*-quinone when exposed. This compound appears to be more stable as a constituent in the crude ethanolic extract. Additionally, high polar compounds which are present in the MeOH fraction could act synergistically with 4-nerolidylcatechol to enhance its significant antioxidant potential. The antioxidant activity of phenols can be attributed to the presence of phenolic groups [19], which are extremely susceptible to oxidation in function of their structures. Besides the presence of highly oxidizable catecholic group, the presence of an unsaturated aliphatic chain can also contribute to the high antioxidant potential of 4-nerolidylcatechol. This compound alone and fractions, rich in phenolic compounds, are able to neutralize free hydroxyl radicals and reactive oxygen species by reducing the oxidative stress that induces DNA damages [20].

Mongelli et al. [25] reported the cytotoxicity of 4-nerolidylcathecol demonstrating that, by a mechanism of inhibition of the activity of topoisomerase I, this substance induces growth inhibition of KB cells. Afterward, the same group has investigated its larvicidal activity, showing that the 4-nerolidylcathecol has a considerable activity [26]. A detailed analysis of the molecular structure of 4-nerolidylcathecol suggests that the catechol moiety would appear to be relevant to the observed cytotoxicity in 4-nerolidylcatechol, while the nerolidyl side chain would appear not to be a necessary structural element for the observed cytotoxicity [24] once 4-nerolidylcatechol can undergo an autooxidation by product of the catechol to yield an *o*-quinone which is probably responsible for the cytotoxic effect observed of both 4-nerolidylcatechol and CH_2_Cl_2_ fraction [27, 28]. Therefore, these mechanisms should contribute to its considerable cytotoxic effect.

## 5. Conclusion

The data presented in this work corroborate the significant antioxidant potential of the crude ethanol extract obtained from the aerial parts of *Pothomorphe umbellata *L. and of its major compound, 4-nerolidylcathecol. Considering its noteworthy antioxidant effect and low cytotoxicity, it must be stated that the crude aqueous-ethanol extract prepared from the aerial parts of *Pothomorphe umbellata *L. and widely used in folklore medicine appears to be a safe and effective natural remedy. 

## Figures and Tables

**Table 1:** Antioxidant effect evaluation of *Pothomorphe umbellata* L.

Experimental samples	Antioxidant effect
	IC_50_ (*μ*g/mL)
*P. umbellata* L. crude extract	1.2 ± 0.2
*P. umbellata* L. CH_2_Cl_2_ fraction	5.9 ± 0.4
*P. umbellata* L. EtOAc fraction	8.0 ± 0.6
*P. umbellata* L. MeOH fraction	4.5 ± 0.3
*P. umbellata* L. sterol fraction	>12.5
4-Nerolidylcatechol	8.6 ± 0.3
Vitamin C	1.7 ± 0.1
Trolox	0.9 ± 0.2

**Table 2:** Cytotoxic effect evaluation of *Pothomorphe umbellata* L.

Experimental samples	Cytotoxic effect
	IC_50_ (*μ*g/mL)
*P. umbellata* L. crude extract	5.3 ± 0.4
*P. umbellata* L. CH_2_Cl_2_ fraction	2.3 ± 0.1
*P. umbellata* L. EtOAc fraction	>10.0
*P. umbellata* L. MeOH fraction	>10.0
*P. umbellata* L. sterol fraction	8.9 ± 0.7
4-Nerolidylcatechol	0.4 ± 0.05
Vitamin C	>10.0
Trolox	>10.0
